# Identification of biomarkers related to CD8^+^ T cell infiltration with gene co-expression network in clear cell renal cell carcinoma

**DOI:** 10.18632/aging.102841

**Published:** 2020-02-20

**Authors:** Jiaxing Lin, Meng Yu, Xiao Xu, Yutao Wang, Haotian Xing, Jun An, Jieping Yang, Chaozhi Tang, Dan Sun, Yuyan Zhu

**Affiliations:** 1 Department of Urology, The First Hospital of China Medical University, Shenyang 110001, Liaoning, China; 2 Department of Reproductive Biology and Transgenic Animal, China Medical University, Shenyang 110001, Liaoning, China; 3 Department of Pediatric Intensive Care Unit, The Shengjing Hospital of China Medical University, Shenyang 110001, Liaoning, China

**Keywords:** clear cell renal cell carcinoma (ccRCC), CIBERSORT, weighted gene co-expression network analysis (WGCNA), CD8+ T cells, CCL5

## Abstract

Clear cell renal cell carcinoma (ccRCC) is an extremely common kind of kidney cancer in adults. Immunotherapy and targeted therapy are particularly effective at treating ccRCC. In this study, weighted gene co-expression network analysis and a deconvolution algorithm that quantifies the cellular composition of immune cells were used to analyze ccRCC expression data from the Gene Expression Omnibus database, and identify modules related to CD8^+^ T cells. Ten hub genes (*LCK*, *CD2*, *CD3D*, *CD3G*, *IRF1*, *IFNG*, *CCR5*, *CD8A*, *CCL5*, and *CXCL9*) were identified by co-expression network and protein-protein interactions network analysis. Datasets obtained from The Cancer Genome Atlas were analyzed and the data revealed that the hub genes were meaningfully up-regulated in tumor tissues and correlated with promotion of tumor progression. After Kaplan-Meier analysis and Oncomine meta-analysis, *CCL5* was selected as a prognostic biomarker. Finally, the experimental results show that reduced expression of *CCL5* decreased cell proliferation and invasion in the ccRCC cell line. Various analyses were performed and verified, *CCL5* is a potential biomarker and therapeutic target which related to CD8^+^ T cell infiltration in ccRCC.

## INTRODUCTION

Renal cell carcinoma (RCC) is one of the most common malignant tumors, ranking seventh among male malignant tumors and tenth among female malignant tumors [1]. RCC accounts for 80% of all kidney cancer, and clear cell renal cell carcinoma is the most common subtype of renal cell carcinoma [2]. Smoking, obesity, and high blood pressure increase the risk of kidney cancer [3]. In recent years, immune checkpoint inhibitors have become the standard for first-line treatment of renal cell carcinoma [4–6]. However, no specific molecular markers have been for immunotherapy of renal cell carcinoma [2]. Therefore, the exploration of immune-related molecular markers is an important focus of renal cell carcinoma research.

RCC are prone to immune infiltration, and the characteristics of tumor microenvironment strongly alter the response to immunotherapy [7]. CD8^+^ T cells contribute to tumor adaptive immunity. Among the immune cells of ccRCC, CD8^+^ T cells account for the largest proportion [8]. In most solid tumors, highly infiltrating CD8^+^ T cells are beneficial to tumor treatment [9–11], but high infiltration of CD8^+^ T cells in RCC is related to bad prognosis [12]. Many studies have explored the immune-related biomarkers of renal cell carcinoma, but the findings cannot be directly applied to actual clinical work. Previous studies have reported that low expression of *CD40* is associated with poor prognosis in patients with RCC [13]. Co-expression of *PD-1* and *Tim-3* was reported to correlate with poor overall survival [14]. However, their studies included less than 50 samples, so the result should be validated in a larger cohort. *CD19* was identified as a surface marker of B cells and can predict the prognosis of metastatic renal cell carcinoma [15]. However, CD8^+^ T cells are more important in tumor adaptive immunity, so it is unclear if *CD19* can guide the immunotherapy of renal cell carcinoma. Therefore, the identification of biomarkers related to CD8^+^ T cell infiltration will facilitate the monitoring of RCC immunotherapy response and the exploration of immune infiltration mechanism.

With the rapid development of bioinformatics technology, many tools have been developed to find biomarkers [16]. Weighted gene co-expression network analysis (WGCNA) is an effective tool that can be used to mine related patterns between genes to identify relevant modules and hub genes for cancer [17]. This algorithm has been widely used to find biomarkers at the transcriptional level [18, 19]. Cell-type Identification by Estimating Relative Subsets of RNA Transcripts (CIBERSORT) is another bioinformatics tool for analysis of gene expression data. This tool quantifies the cellular composition of immune cells using a deconvolution algorithm [20]. This algorithm has been successfully used to approximate the level of immune cell infiltration in various cancers, such as prostate cancer [21] and kidney cancer [8].

To explore the effect of the tumor microenvironment and identify potential biomarkers of ccRCC, WGCNA was performed using ccRCC gene expression data. The T-cell compositions of samples were calculated using the CIBERSORT algorithm. We then identified important modules and hub genes related to CD8^+^ T cell infiltration levels, and the immune and clinical features of these genes were verified by database analysis. Prognostic biomarkers were then identified and verified. This is the first utilization of WGCNA to identify CD8^+^ T cell-related biomarkers of ccRCC.

## RESULTS

### RNA expression data

**Figure 1:**
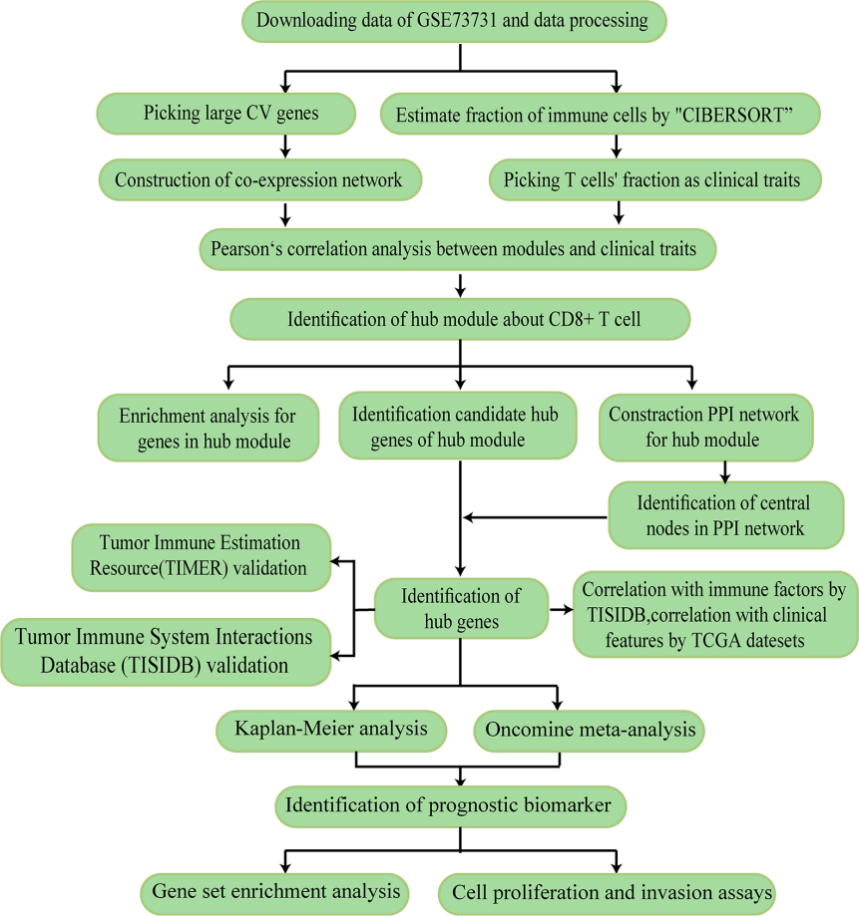
**The workflow of the study.**

The research strategy is presented in Figure 1.

We obtained RNA expression data for 265 ccRCC samples Gene Expression Omnibus (GEO) database. All data for tumor samples in the dataset were obtained, and 4411 genes with Coefficient of variation values greater than 0.1 were selected for additional analysis (Supplementary Table 1).

### Evaluation of tumor-infiltrating immune cells (TIICs)

CIBERSORT is an analytical algorithm that analyzes RNA expression data to assess the abundance of different cell subtypes for each sample. The fractions of 22 TIICs were calculated by using the R package “CIBERSORT”. Then, the fractions of seven subtypes of T cells in every sample were selected as trait data of WGCNA (Supplementary Table 2).

### Gene co-expression network of ccRCC

**Figure 2:**
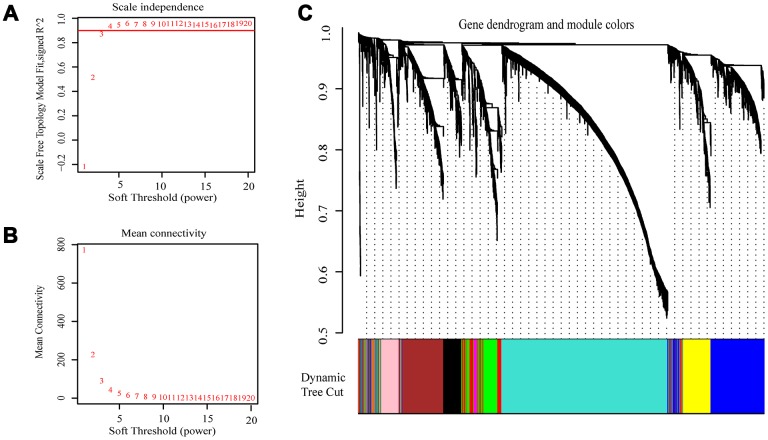
**Selection of the appropriate beta value to construct a hierarchical cluster number.** (**A**) Analyze the scale-free fit index of the 1-20 soft threshold power (β). (**B**) Analyze the average connectivity of 1-20 soft threshold power. (**C**) Genes are grouped into various modules by hierarchical clustering, and different colors represent different modules.

The expression values of the 4411 genes were used to construct a co-expression network using the R package “WGCNA” We calculated average linkage and Pearson’s correlation values to cluster the samples of GSE73731 (Supplementary Figure 1). To build a scale-free network, we picked β = 3 (scale free R^2^ = 0.8723676) as the soft-thresholding power (Figure 2A, 2B). A hierarchical clustering tree was constructed using dynamic hybrid cutting. Each leaf on the tree represents a single gene, and genes with similar expression data are close together and form a branch of the tree, representing a gene module. Nine modules were generated (Figure 2C).

### Identification of hub modules and enrichment analysis

**Figure 3:**
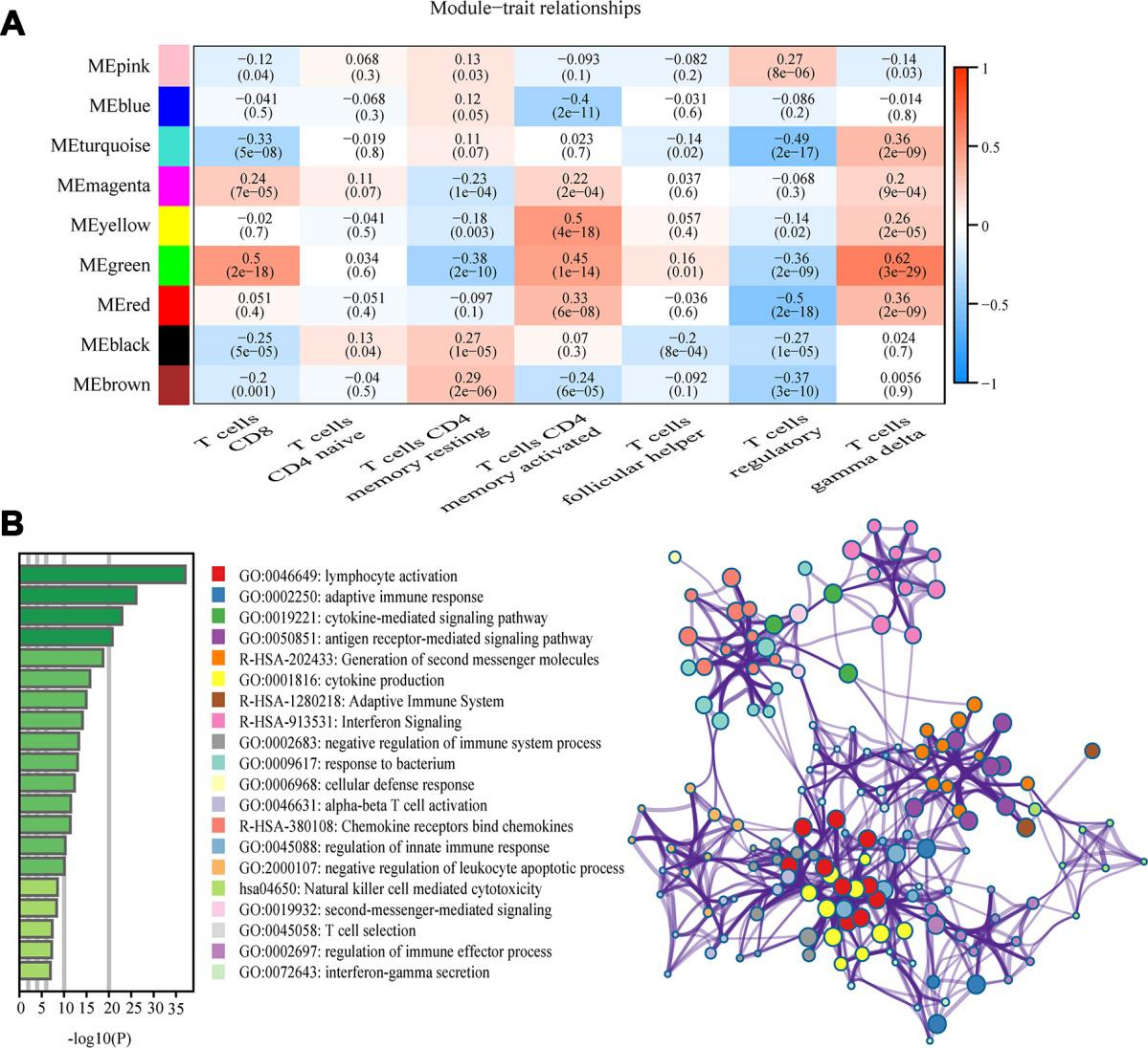
**Key modules and feature notes.** (**A**) Heatmap shows correlations of module eigengenes with T-cell infiltration. (**B**) The first 20 enriched terms are shown as a bar chart on the left. The network diagram on the right is constructed with each enrichment term as a node and the similarity of the node as the edge. Nodes with the same cluster ID are the same color.

Among the nine module, the green module was highly correlated to T cells CD8 (CD8^+^ T cells) (R^2^=0.5, P=2e-18), T cells CD4 memory activated (R^2^=0.45, P=1e-14), and T cells gamma delta (R^2^=0.62, P =3e-29), and the yellow module showed higher correlation with activated T cells CD4 memory activated (R^2^=0.5, P=4e-18; Figure 3A). The correlation between other modules and T cells was less than 0.5. We were interested specifically in the CD8^+^ T cells, so focused on the green module that showed correlation with CD8^+^ T cells was identified as a hub module. Genes included in this module were next analyzed using the web tool “Matascape” for pathway and process enrichment analysis. The 20 highest enrichment terms were all immune-related terms (Figure 3B), and the three most highly enriched terms were Lymphocyte Activation, Adaptive Immune Response, and Cytokine-mediated Signaling Pathway (Supplementary Table 3).

### Identification and validation of hub genes

**Figure 4:**
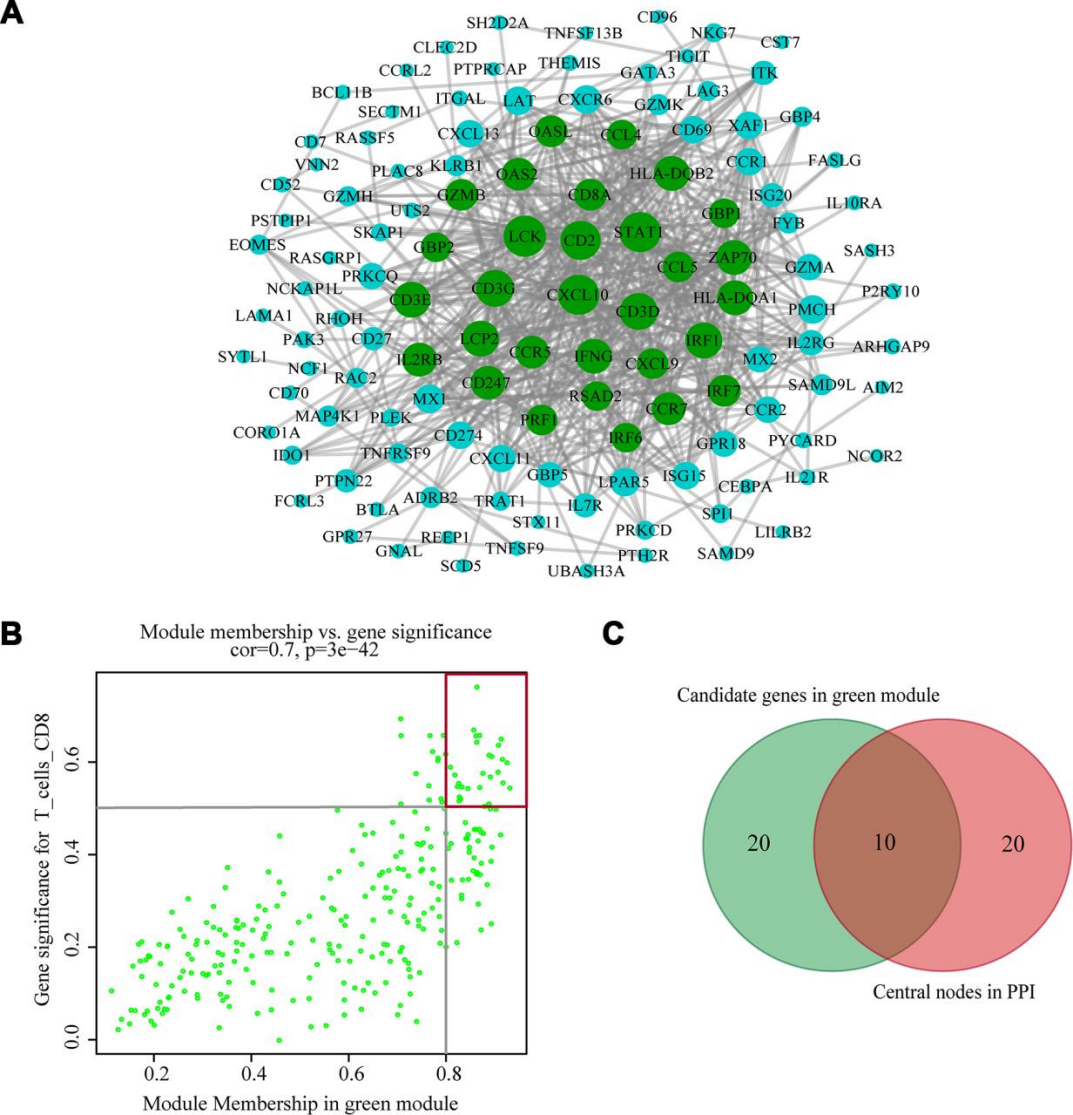
**Identification of hub genes.** (**A**) PPI network of genes from the green module. The higher the number of connected nodes, the larger the size of the node. The green nodes represent a central node with more than 15 connections. (**B**) A scatter plot of the genes in the green module. Each green dot represents a gene, and dots within the red box indicate genes of Module Membership > 0.8 and Gene Significance > 0.5. (**C**) Hub genes were selected based on overlap between PPI and co-expression networks.

**Figure 5:**
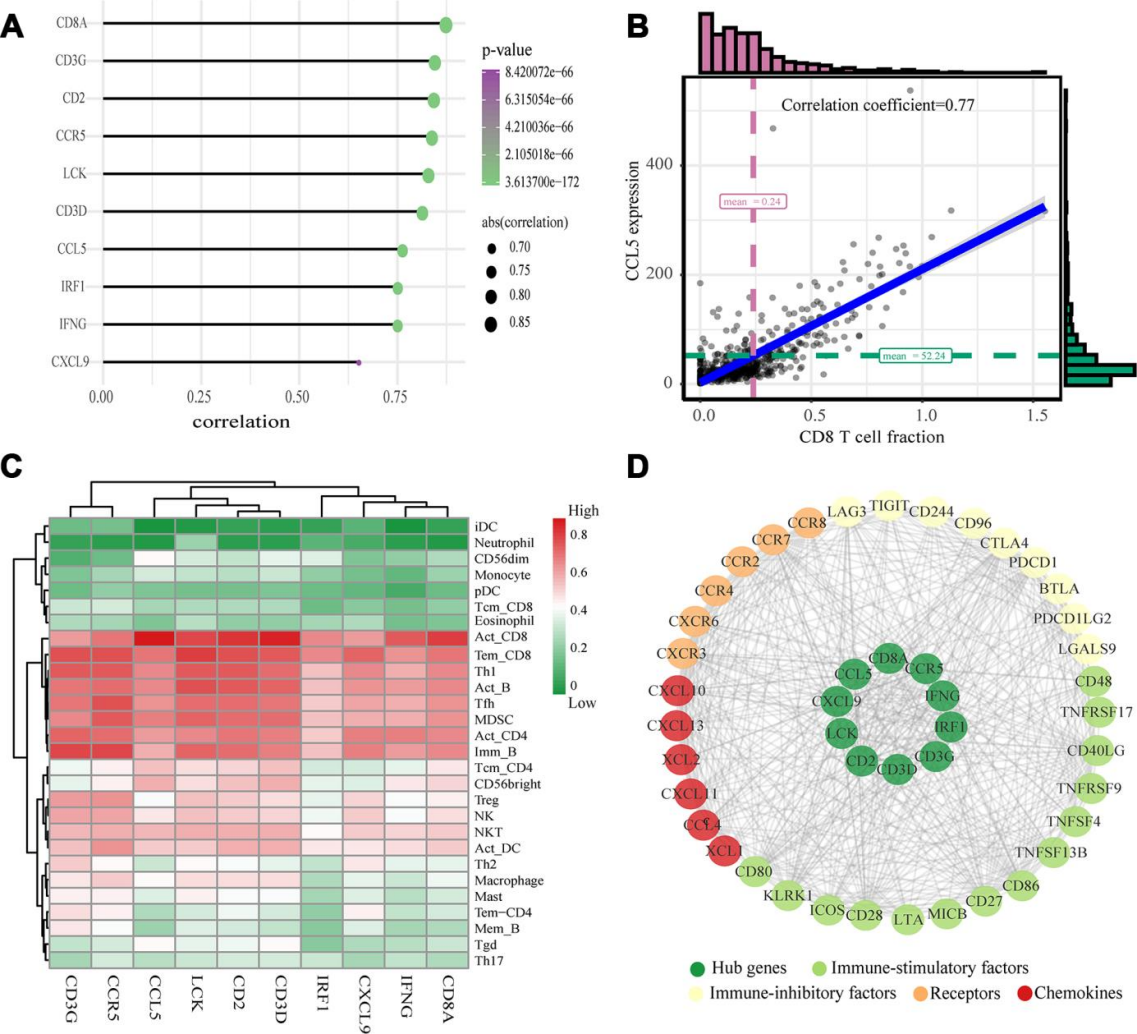
**Validation of hub genes and PPI map construction.** (**A**) Relationship between 10 hub genes expression and CD8^+^ T cell infiltration level; P < 0.05 is considered statistically significant. (**B**) Scatter plot of *CCL5* expression and CD8^+^ T cell infiltration level. (**C**) The heat map shows the correlation between the ten identified hub genes and the TIICs from the TISIDB database. The redder color indicates a higher correlation, and greener color indicates a lower correlation. (**D**) Protein-protein interaction map of the ccRCC immune microenvironment.

The highly connected genes of the module were investigated as potential key factors related to CD8^+^ T cell infiltration level. According to the cut-off standard (Module-Membership > 0.8 and Gene-Significance > 0.5), 30 genes were selected as candidate hub genes (Figure 4B). From the protein-protein interactions (PPI) network, cut-offs of reliability > 0.7 and connectivity > 15 (node/edge) were applied to identify 30 genes as central nodes, and we visualized these results using Cytoscape (Figure 4A). Ten genes were selected in both analyses designated as hub genes (*LCK*, *CD2*, *CD3D*, *CD3G*, *IRF1*, *IFNG*, *CCR5*, *CD8A*, *CCL5* and *CXCL9*) (Figure 4C). To investigate the relationship between these hub genes and CD8^+^ T cells, we analyzed the expression data for these genes in the TIMER database. The results showed positive correlation of the expression values of the 10 genes with the infiltration levels of CD8^+^ T cells (correlation coefficient of at least 0.75 for all genes except *CXCL9*) (Figure 5A). As an example, we show a scatter plot of *CCL5* expression and CD8^+^ T cell infiltration level in Figure 5B. We next queried the TISIDB database to obtain the Spearman correlation values between abundance of tumor-infiltrating lymphocytes and gene expression. The results show a positive correlation between hub genes and tumor-infiltrating lymphocytes. The correlation values were highest for Activated CD8^+^ T cell (Act CD8) and Effector memory CD8^+^ T cell (Tem CD8) (Figure 5C). These analyses verified the identified hub genes as strongly associated with the CD8^+^ T cell infiltration level and playing significant roles in the immune microenvironment.

### Determination of immune and clinical characteristics

**Figure 6:**
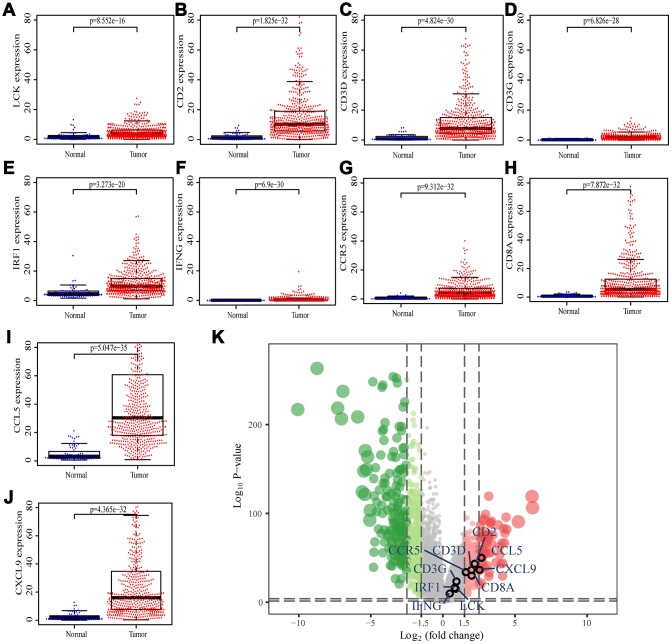
**Differential expression of the hub genes in transcriptional data of TCGA.** (**A**) *LCK*, blue dots represent normal tissue and red dots represent tumor tissue. The y-axis shows the expression value of the gene. (**B**) *CD2*. (**C**) *CD3D*. (**D**) *CD3G*. (**E**) *IRF1*. (**F**) *IFNG*. (**G**) *CCR5*. (**H**) *CD8A*. (**I**) *CCL5*. (**J**) *CXCL9*. (**K**) The volcano plot of differentially expressed genes. Red dots indicate overexpression genes, green dots indicate low expression genes, and black circles represent hub genes.

**Figure 7:**
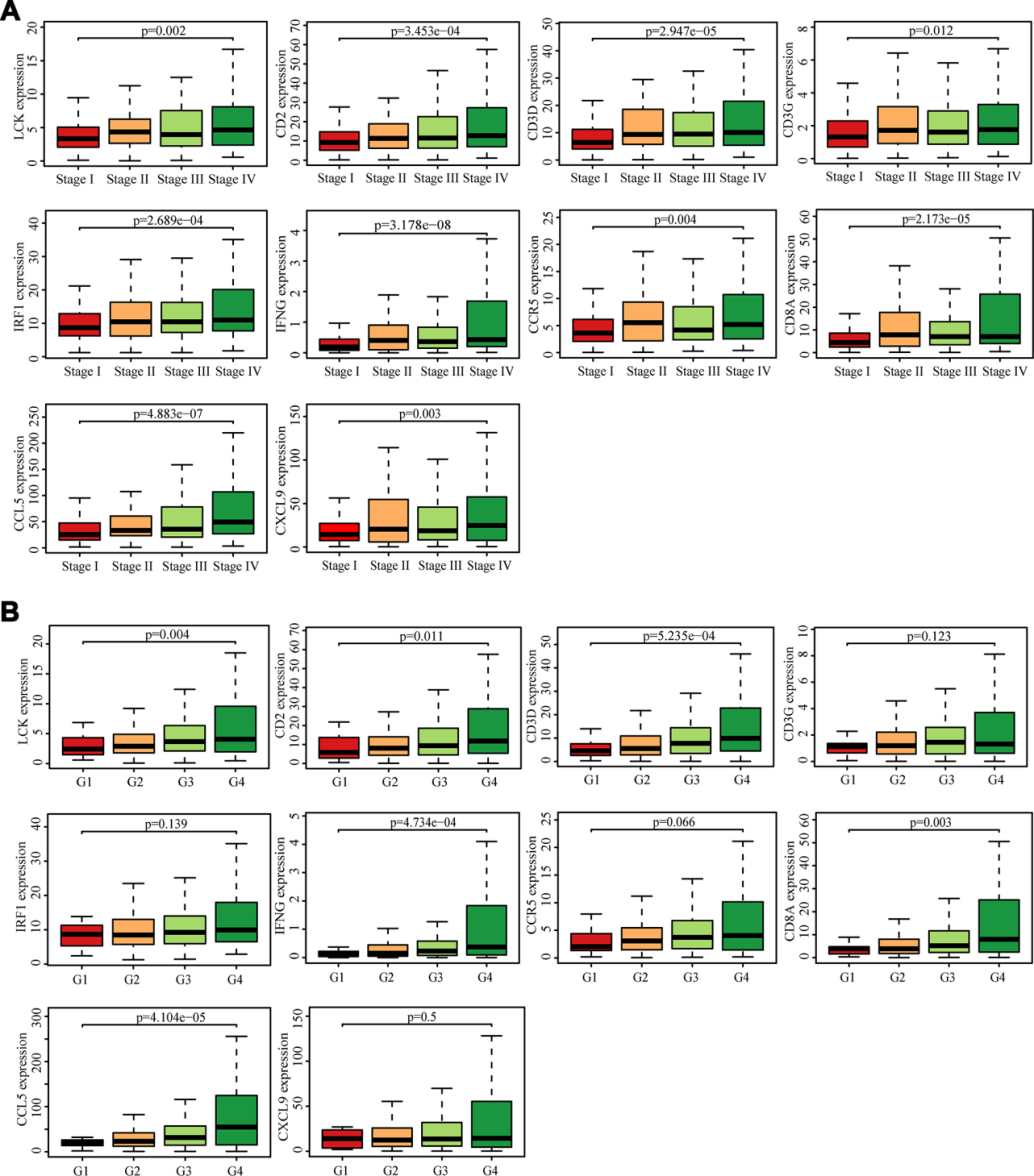
**Analysis of hub genes and clinical indicators in the TCGA dataset.** (**A**) Box plot of the hub genes for different pathological stages. (**B**) Box plot of the hub genes for different tumor grades.

We searched for the Spearman correlations of expression of these 10 hub genes with the expression of immune factors in TISIDB database, including immune-inhibitory factors, immune-stimulatory factors, chemokines, and receptors. These correlations are presented as heat maps (Supplementary Figure 2). We identified 38 immune-related factors with average correlations with the 10 hub genes greater than 0.5. We constructed an immune infiltration interaction network based on the 10 hub genes and the 38 immune-related factors to explore the infiltration mechanism of CD8^+^ T cells using the STRING database. The results were imported into Cytoscape for visualization (Figure 5D). We next obtained the expression levels of the 10 genes of ccRCC from the The Cancer Genome Atlas (TCGA). The expression levels of these genes were higher in tumor tissues than in normal tissues (P < 0.05) based on Wilcoxon signed-rank test (Figure 6A–6J). Volcanic map also showed that the expression of 10 genes in tumor tissues was higher than that in normal tissues (Figure 6K), with corrected p values of all genes less than 0.05, indicating statistical significance. The fold changes of *CCL5* and *CXCL9* were more than 2.5-fold higher in tumor tissues than the levels in normal tissues (Supplementary Table 4). The relationships between hub genes and pathological stages are shown by boxplot (Figure 7A). Expression levels of all hub genes show significant differences in pathological stages (p < 0.05) and showed an upward trend with increased stages. Finally, we investigated the connection between tumor grades and hub genes (Figure 7B), in which *LCK*, *CD2*, *CD3D*, *IFNG*, *CD8A*, and *CCL5* showed significant correlation with different tumor grades (p < 0.05), with grade increase corresponding to increased gene expression. Although no significant difference was detected for *CD3G*, *IRF1*, *CCR5*, and *CXCL9*, the gene expression level showed an upward trend with increased tumor grades.

### Identification of prognostic biomarker

**Figure 8:**
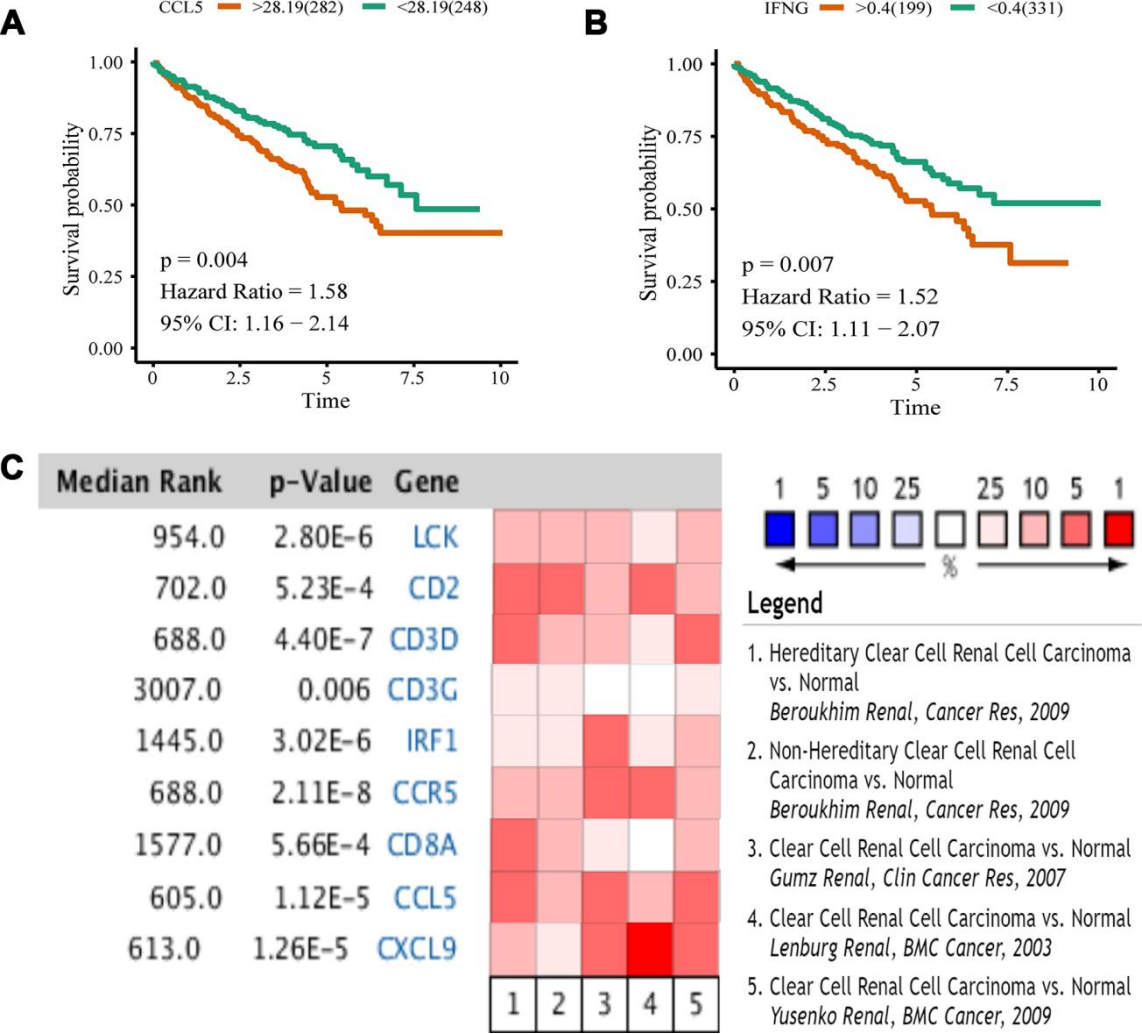
**Kaplan-Meier analysis and Oncomine meta-analysis.** (**A**) The overall survival analysis of *CCL5*. (**B**) The overall survival analysis of *IFNG*. (**C**) A meta-analysis of gene expression from Oncomine datasets. Colored squares represent the median of genes (relative to normal tissue) in five analyses. Red represents overexpression, blue represents low expression. This P value gives the average rank analysis.

We analyzed 10 hub genes by Kaplan-Meier analysis, and only the results of *CCL5* and *IFNG* were statistically significant (p < 0.05). For these two genes, the survival prognosis of patients with high expression was poor (Figure 8A, 8B). To validate differential expression of 10 genes in tumor and normal tissues, we used Oncomine to perform a meta-analysis of five analyses using four data sets, all of which included both tumor tissues and normal controls for 10 genes (Figure 8C). Data for *IFNG* were not included in these data sets, so we obtained meta-analytical results for the other nine genes. *LCK*, *CD2*, *CD3D*, *CCR5*, *CCL5*, and *CXCL9* have Median Rank values less than 1000, *CD3G* is 3007.0, *IRF1* is 1445.0, and *CD8A* is 1577.0. The results show these genes exhibit significant overexpression in tumor tissues which was consistent with the TCGA datasets analyses. Through Kaplan-Meier and Oncomine meta-analysis, we selected *CCL5* as a prognostic biomarker for further analysis.

### Gene set enrichment analysis of *CCL5*

**Figure 9:**
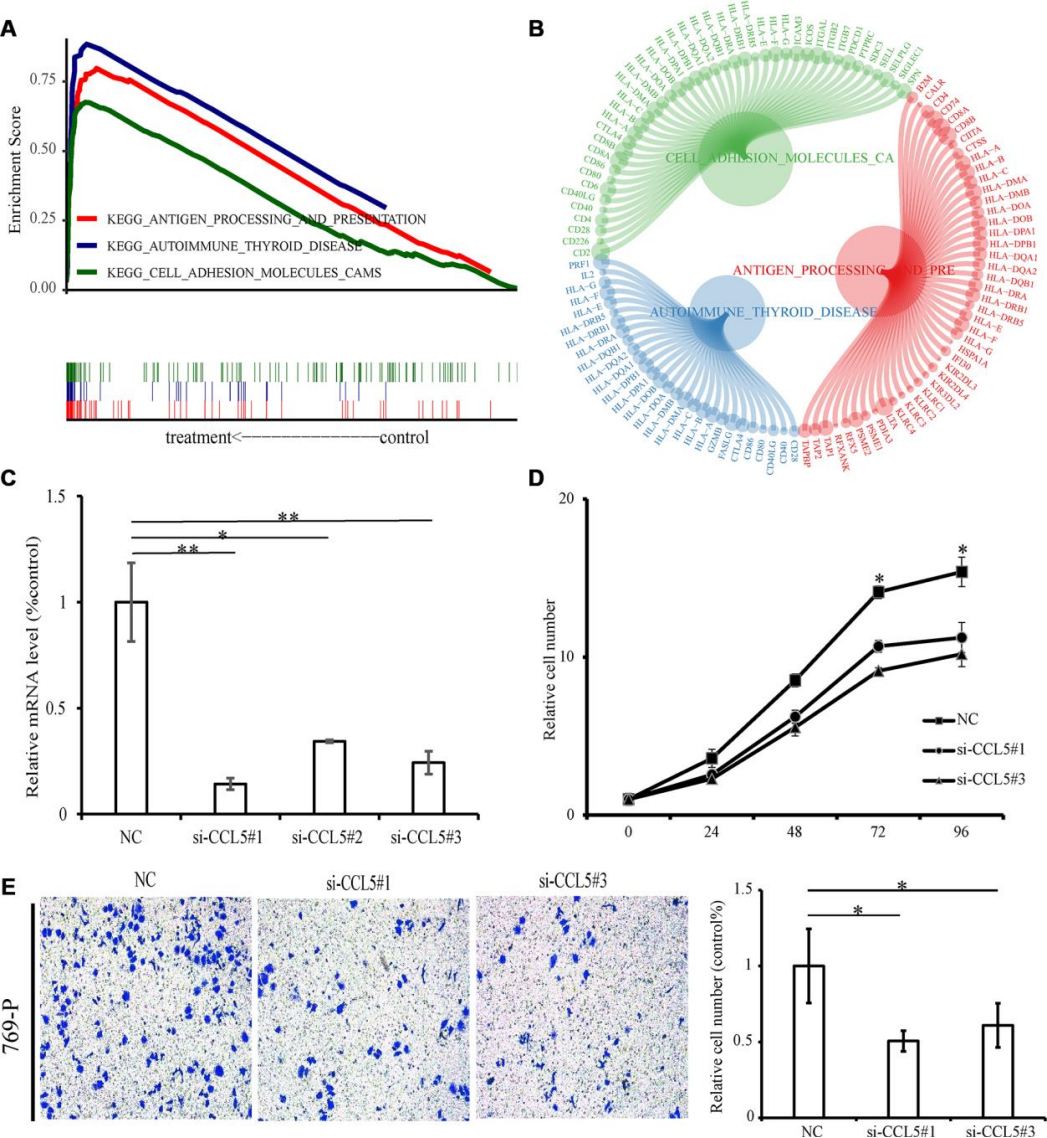
**GSEA and experiment of *CCL5*.** (**A**) The above picture shows the enrichment fractional broken line of the three pathways, and the lines in the lower figure correspond to the genes of each pathway. (**B**) The circle diagram shows three enrichment pathways and the core genes that play a role in the enrichment process. The larger the circle corresponding to each gene, the larger the rank metric score value. (**C**) The relative mRNA levels of normal control and si-*CCL5* of 769-P cells. (**D**) The results of CCK-8 assay showed decreased proliferation ability of 769-P cells when treated with si-*CCL5*. (**E**) The invasion assay results showed decreased invasion ability of 769-P cells treated with si-*CCL5*.

According to *CCL5* expression median value, ccRCC samples from TCGA were divided into high expression group and low expression group for pathway gene set enrichment analysis. The enrichment results showed that immune-related pathways were enriched in the high expression group, with a total of 23 pathways statistically significantly enriched (p-value < 0.05, q-value < 0.05). The three most enriched pathways were “Antigen procession and presentation”, “Cell adhesion molecules cams”, and “Autoimmune thyroid disease” (Figure 9A, 9B, Supplementary Table 5). There were no significantly enriched pathways for the low expression group.

### Knockdown of *CCL5* significantly inhibited cell proliferation and invasion

Because *CCL5* is overexpressed in ccRCC and is related to poor prognosis, we next conducted functional experiments to explore the potential biological function of *CCL5*. First of all, we used small interfering RNAs to lower expression levels of *CCL5* in the renal cell carcinoma cell line 769-P (Figure 9C) and evaluated the cell proliferation ability by Cell Counting Kit-8 (CCK-8). The results showed decreased cell line proliferation ability after *CCL5* knockdown (Figure 9D). Finally, the invasion ability of the cell line decreased significantly after *CCL5* knockdown (Figure 9E).

## DISCUSSION

Clear cell renal cell carcinoma is the main histological subtype of RCC, and has relatively poor prognosis [22]. The successful application of immune checkpoint inhibitors in ccRCC has increased interested in exploring the potential targeting of specific immune-related factors for immunotherapy [7]. CD8^+^ T cells plays a central role in immunotherapy. In this study, we identified 10 hub genes whose expression correlated to CD8^+^ T cell infiltration level, suggesting a potential mechanism through which these genes promote the progress of ccRCC. Of the identified 10 genes, *CCL5* was identified as a potential prognostic biomarker and target. Significant progress has been made in the study of the molecular basis of ccRCC by using animal tumor models, *in-vitro* cell lines, and clinical samples. However, the complexity of the ccRCC microenvironment requires further analysis and larger datasets. We used gene expression matrix to construct the co-expression network and calculate the infiltration level of T cells, and correlations were determined to identify the genes most related to CD8^+^ T cells. The gene enrichment analysis of the selected module shows that it is a highly immune-related module. The most highly connected genes in the co-expression network and the protein-protein network were considered hub genes (*LCK*, *CD2*, *CD3D*, *CD3G*, *IRF1*, *IFNG*, *CCR5*, *CD8A*, *CCL5* and *CXCL9*). Querying the relationship between these 10 genes and immune cells in the TIMER/TISIDB database revealed positively correlated expression of these genes with immune cells, particularly CD8^+^ T cells. We used hub genes and related immune factors to construct a CD8^+^ T cell infiltration network using the TISIDB and STRING databases as a strategy to explore the immune mechanism of ccRCC. The TCGA datasets were used to observe the differential expression and clinical characteristics of the selected 10 genes. The results showed overexpression of the 10 genes in tumor tissue, indicating potential use as biomarkers. Expression correlated with increase of tumor stage and grade are especially important for potential prognostic factors. These 10 hub genes can explain why the prognosis of ccRCC is poor in the case of highly infiltrated CD8^+^ T cells. We performed Kaplan-Meier analysis for the 10 hub genes. Only the results of *CCL5* and *IFNG* were statistically significant, and the prognosis was poor when these genes were highly expressed. The Oncomine database was used to perform a meta-analysis, and the difference between cancer tissue and normal tissue of *CCL5* expression was the most significant. Combined with these two analyses, *CCL5* was selected as the best potential biomarker for the detection and prediction prognosis of renal cell carcinoma.

Chemokine ligand 5 (*CCL5*) belongs to the CC chemokine family, which acts mainly by binding to its corresponding chemokine receptor. Several studies have focused on the effect of *CCL5* on tumors, finding that *CCL5* can significantly promote tumor growth, metastasis [23], angiogenesis [24, 25], and immune escape [26, 27]. *CCL5* is expressed not only in immune cells, but also in tumor cells [28]. The expression of *CCL5* in breast cancer cells promotes the proliferation and invasion of breast cancer cells through an autocrine pathway [29, 30]. *CCL5* also promotes the progression of melanoma [31] and pleomorphic glioma [32]. However, there has been no experimental study of the effect of *CCL5* on the proliferation and invasion of ccRCC cells. The experimental results show that si-*CCL5* can decrease the proliferation and invasion ability of ccRCC cells, indicating that *CCL5* could be utilized as a therapeutic target.

In short, this study is the first attempt to use WGCNA and CIBERSORT algorithms to identify potential CD8^+^ T cell related biomarkers of ccRCC. Ten hub genes were identified which were overexpression in tumor and promoting tumor progression. Through multiple verification of bioinformatics and experiments, *CCL5* was identified as a potential biomarker and target for clear cell renal cell carcinoma immunotherapy. However, this study has certain limitations. Additional sample data are needed to verify these results and the specific mechanism of *CCL5* in ccRCC requires further investigation.

## MATERIALS AND METHODS

### Gene expression data and processing

We downloaded ccRCC RNA expression data from the Gene Expression Omnibus (GEO, http://www.ncbi.nlm.nih.gov/geo/), which contains data related to 265 samples [33]. The dataset of GSE73731 was obtained using the platform Affymetrix Human Genome U133 Plus 2.0 Array (HG U133 Plus 2.0). We used the R package “limma” [34] to normalize the RNA-sequencing data. Small variation of gene expression data often represents noise, so we used Coefficient of Variation values to select the most variant genes, which were then used to construct the network.

### Evaluation of tumor-infiltrating immune cells

In this study, we used the R package “CIBERSORT” to estimate the fraction of immune cells of GSE73731 samples. Specifically, the CIBERSORT algorithm was used to calculate the fractions of the 22 types of TIICs [20]. CIBERSORT is considered better to previous deconvolution methods for analysis of unknown mixture content and noise. This algorithm can be used to statistically estimate the relative proportions of cell subpopulations from complex tissue expression profiles, making it a useful tool to estimate the abundances of special cells in mixed tissue.

### Co-expression network construction

Expression values of 4411 genes were used to construct a weight co-expression network using the R package “WGCNA” [17]. First, based on the Pearson’s correlation value between paired genes, the expression levels of individual transcripts were converted into a similarity matrix. Next, the similarity matrix was transformed to an adjacency matrix, as calculated by amn = |cmn| β (cmn = Pearson’s correlation between paired genes; amn = adjacency between paired genes). Parameter β can improve strong correlations and decrease weak correlations between genes. The adjacency matrix was then converted into a topological overlap matrix when the power of β = 3. To categorize genes with similar expression patterns into different modules, we applied a dynamic hybrid cutting method, using a bottom-up algorithm with a module minimum size cutoff of 30.

### Construct module trait relationships

Module eigengenes were used to perform component analysis of each module. We calculated the correlation between module eigengenes and the infiltration level of T cells to determine the significance of modules by Pearson test. An individual module was considered significantly correlated with T cells when p < 0.05. We selected the interest T cell subtype and module with the highest correlation coefficient and defined that as a hub module.

### Pathway and process enrichment analysis

To determine the function of genes in the identified hub module, we used the web tool “Metascape” (http://metascape.org) for pathway and process enrichment analysis [35]. The tool displays the first 20 enriched terms as a bar graph. In order to further explore the relationship between terms, terms with similarity greater than 0.3 are connected by edges and presented as a network graph.

### Identification of hub genes

We selected candidate hub genes based on the modular connectivity and clinical traits relationship of each gene in the hub module. Module connectivity is defined as the absolute value of the Pearson’s correlation between genes (Module Membership). Clinical trait relationship is defined as the absolute value of the Pearson’s correlation between each gene and the trait (Gene Significance). We set the Module-Membership > 0.8 and the Gene-Significance > 0.5 for candidate hub genes. Meanwhile we selected all genes in the hub module and used the Search Tool for the Retrieval of Interacting Genes (STRING; https://string-db.org/) database to construct PPI network and looked for central nodes [36]. Genes with node connectivity > 15 were considered central nodes. We used Cytoscape to present the network (https://cytoscape.org/) [37]. We did Venn analysis to compare candidate hub genes and central nodes in the PPI network using the online tool (http://bioinformatics.psb.ugent.be/webtools/Venn/).

### Validation of hub genes

We used two immune-associated databases that are based on TCGA to validate these hub genes, as described below. First, we obtained the content of CD8^+^ T cells in each sample of ccRCC based on data in the Tumor Immune Estimation Resource (TIMER; https://cistrome.shinyapps.io/timer/) [38]. Spearman correlations between the infiltration level of CD8^+^ T cells and the expression of hub genes were calculated, and the results were compared using the R package “ggstatsplot”. Second, the Tumor Immune System Interactions Database (TISIDB; http://cis.hku.hk/TISIDB) [39] was then searched to determine Spearman correlations between the hub genes and TIICs. We present these results as a heat map constructed using the R package “pheatmap”.

### Immune and clinical characteristic identification

Spearman correlations between hub genes and different immune factors were obtained from the TISIDB database, which includes immune-inhibitory and immune-stimulatory factors, chemokines, and receptors. We then constructed a heat map by using the R package “heatmap”. Immune factors related to hub genes with average correlation coefficients greater than 0.5 were picked to construct a network using STRING and Cytoscape [37]. To explore the clinical characteristic of hub genes, RNA expression data and clinical data of ccRCC were obtained from The Cancer Genome Atlas (TCGA; https://cancergenome.nih.gov/). The R packages “limma” and “beeswarm” were used to construct a scatter differential diagram of the data. The statistical significance of differences in expression between normal and tumor samples was analyzed using the Wilcoxon signed-rank test. The differences of all coding genes were analyzed by R package “limma", and the volcano map was drawn using the R package “ggplot2”. Finally, a boxplot was constructed to display the relationship between genes and clinical features, and the statistical significances were analyzed by the Kruskal-Wallis test.

### Kaplan-Meier analysis and Oncomine mata-analysis

We found best separation through the R package “survminer” to divide the patients into high and low expression groups, such grouping minimizes the p value of the survival curve. Then took Kaplan-Meier analysis for the groups by R package “survival”. To validate the expression patterns of the hub genes, four independent microarray datasets were used from the Oncomine Cancer Microarray database (Oncomine, https://www.oncomine.org) to perform meta-analysis [40–43].

### Gene set enrichment analysis (GSEA)

GSEA is a computational method used to determine whether a set of basically defined gene sets exhibits statistically significant differences between two biological states [44]. According to the median value of gene expression, the samples were divided into two groups, and “c2.cp.kegg.v7.0.symbols” gene set enrichment analysis was carried out, with p-value < 0.05 and q-value < 0.05 as indicative of statistical significance. The enrichment pathway was visualized using the R packages “ggplot2” and “clusterProfiler”. In the circle diagram, we only show the core genes of the enrichment process, and the size of the dots represent the Rank metric score, which indicates the amount of sequencing value of the gene.

### Cell culture and siRNA-PTEN construction

The 769-P cells were cultivated in DMEM with 10% of fetal calf serum, 100 U/ml penicillin and 100 μg/ml streptomycin. The cells were cultured at 37 °C, with 5% CO_2_. The CCL5-310 siRNA sequences were as follows: 5’-GCUGAACAAGGGAAGCUUTT-3’ 5’-AAGCUU GCCCUUGUUCAGCTT-3’. The CCL5-286 siRNA sequences were: 5’-GCAGGAUUUCCUGUAUG ACTT-3’ and 5’-GUCAUACAGGAAAUCCUGCTT-3’. The CCL5-240 siRNA sequences were: 5’-UC GUCCACAGGUCAAGGAUTT-3’ 5’-AUCCUUGAC CUGUGGACGATT-3’

### RNA extraction and quantitative RT-PCR

RNA samples were extracted from cells with the FastPure Cell/Tissue Total RNA Isolation kit (Vazyme Biotech) and they were reverse-transcribed by HiScript III RT SuperMix for qPCR (Vazyme Biotech). To determine the relative transcript level, PCR was quantified in real-time using a LifeECO PCR machine (BIOER Technology Co, Ltd). SYBR Green was used as the fluorophore. The CCL5 primers sequences were as follows: 5′-CTC ATTGCTACTACTGCCCTCTGCGCTCCTGC-3′ and 5′GCTCATCTCCAAAGAGTTGATGTACTC -3′. The PCR parameters were: 95 °C 5min, followed by 50 cycles of 30 s and 1 min at 60 °C. Each sample was measured in three independent reactions. The threshold values of each sample/primer were determined, and the average error and standard error were calculated. Melting curve analysis was performed, and the mRNA expression levels were normalized against that of β-actin.

### Cell proliferation analysis and invasion experiment

769-P cells were cultivated in five 96-well cell culture plates (1500 cells/well) respectively for 5 days. A volume of 10 μl of CCK-8 solution was added to each well in a plate at an interval of 24 h. The cells were cultures for 3 h in 37 °C, and then a photometry test was performed at wavelength of 450 nm. The 769-P cells were then transferred into the top layer holes in a basement membrane plate with wells containing the medium without serum. The lower cavity was filled with 12% fetal calf serum medium to allow chemical attraction. The medium was removed after 12-hour incubation and the implant in the upper cavity was discarded. Cells invading the lower cavity were fixed with 700 ml of 4% PFA and stained with crystal for 1 h to visualize. The cells in the lower cavity were counted and normalized to the number in control conditions to measure the relative invasion capacity.

## Supplementary Material

Supplementary Figures

Supplementary Table 1

Supplementary Table 2

Supplementary Table 3

Supplementary Table 4

Supplementary Table 5
