# Supplementary material for: Identification of biomarkers related to CD8+ T cell infiltration with gene co-expression network in clear cell renal cell carcinoma
Source: Aging (Albany NY). 2020 Feb 20;12(4):3694–712. doi: 10.18632/aging.102841 (PMC7066925; doi:10.18632/aging.102841)
Supplement: Supplementary Figures [file aging-12-102841-s005..pdf]

SUPPLEMENTARY FIGURES

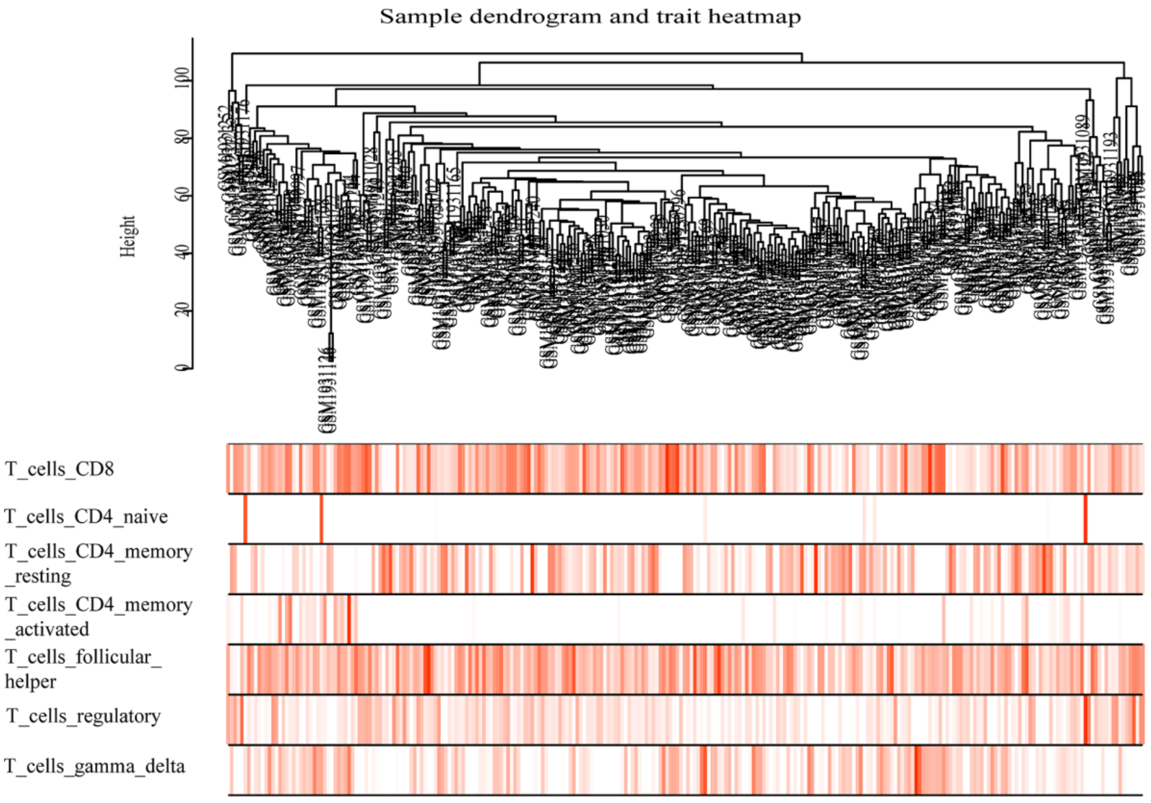

Supplementary Figure 1. Sample clustering (GSE73731): sample dendrogram and trait indicator. In the heat map, the darker the color, the higher the degree of infiltration of the seven kinds of T cells.

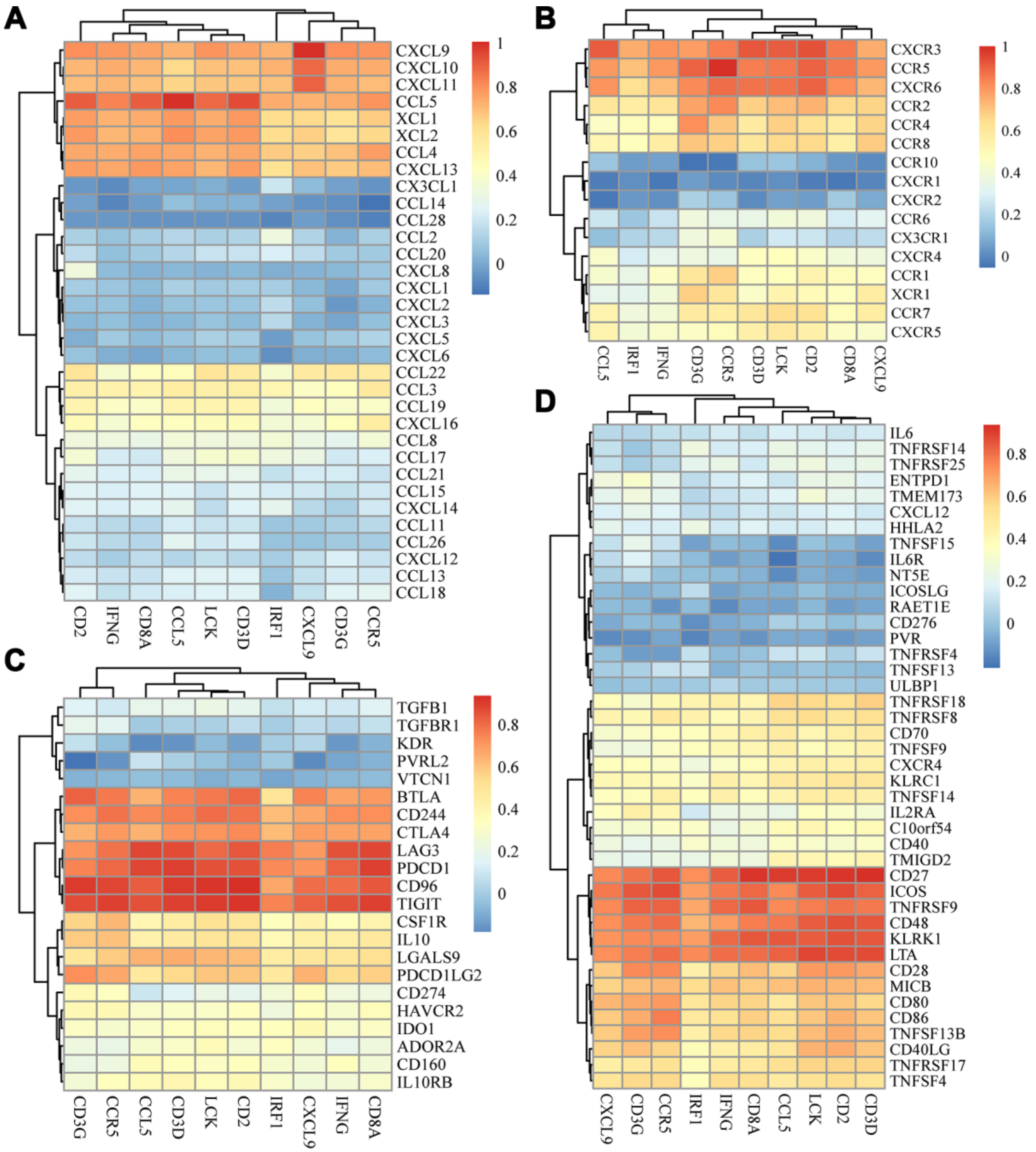

Supplementary Figure 2. Heat map of the correlation between immune factors and hub genes. (**A**) Chemokines. (**B**) Receptors. (**C**) Immune-inhibitory factors. (**D**) Immune-stimulatory factors.
