# Supplementary material for: Identification of biomarkers related to CD8+ T cell infiltration with gene co-expression network in clear cell renal cell carcinoma
Source: Aging (Albany NY). 2020 Feb 20;12(4):3694–712. doi: 10.18632/aging.102841 (PMC7066925; doi:10.18632/aging.102841)
Supplement: Supplementary Table 4 [file aging-12-102841-s004..pdf]

Supplementary Table 4. The result of differential analysis of gene expression between normal tissue and tumor tissue by R package "limma".

Gene	logFC	AveExpr	t	P.Value	adj.P.Val	B
CCL5	2.761844	4.760074	16.69318	8.34E-52	1.82E-50	106.7216
CD2	2.28705	3.233462	15.32089	4.60E-45	7.75E-44	91.25291
CXCL9	2.625227	3.859447	13.8642	3.42E-38	4.27E-37	75.49697
CD3D	2.063852	2.945952	13.82764	5.04E-38	6.23E-37	75.11169
CCR5	1.667775	2.207914	13.2961	1.32E-35	1.44E-34	69.57093
CD8A	2.076911	2.644136	12.42316	9.51E-32	8.71E-31	60.73685
CD3G	1.022994	1.294262	10.84224	3.62E-25	2.40E-24	45.68733
IRF1	0.899948	3.35192	8.877243	7.60E-18	3.42E-17	28.98526
LCK	0.966669	2.169819	8.614633	6.00E-17	2.55E-16	26.94321
IFNG	0.555027	0.53599	6.865693	1.63E-11	5.02E-11	14.62135
